# The Deoxyhypusine Synthase Mutant *dys1-1* Reveals the Association of eIF5A and Asc1 with Cell Wall Integrity

**DOI:** 10.1371/journal.pone.0060140

**Published:** 2013-04-01

**Authors:** Fabio Carrilho Galvão, Danuza Rossi, Wagner da Silva Silveira, Sandro Roberto Valentini, Cleslei Fernando Zanelli

**Affiliations:** Department of Biological Sciences, Univ Estadual Paulista – UNESP, Araraquara-Saõ Paulo, Brazil; Universidade de Sao Paulo, Brazil

## Abstract

The putative eukaryotic translation initiation factor 5A (eIF5A) is a highly conserved protein among archaea and eukaryotes that has recently been implicated in the elongation step of translation. eIF5A undergoes an essential and conserved posttranslational modification at a specific lysine to generate the residue hypusine. The enzymes deoxyhypusine synthase (Dys1) and deoxyhypusine hydroxylase (Lia1) catalyze this two-step modification process. Although several *Saccharomyces cerevisiae* eIF5A mutants have importantly contributed to the study of eIF5A function, no conditional mutant of Dys1 has been described so far. In this study, we generated and characterized the *dys1-1* mutant, which showed a strong depletion of mutated Dys1 protein, resulting in more than 2-fold decrease in hypusine levels relative to the wild type. The *dys1-1* mutant demonstrated a defect in total protein synthesis, a defect in polysome profile indicative of a translation elongation defect and a reduced association of eIF5A with polysomes. The growth phenotype of *dys1-1* mutant is severe, growing only in the presence of 1 M sorbitol, an osmotic stabilizer. Although this phenotype is characteristic of Pkc1 cell wall integrity mutants, the sorbitol requirement from *dys1-1* is not associated with cell lysis. We observed that the *dys1-1* genetically interacts with the sole yeast protein kinase C (Pkc1) and Asc1, a component of the 40S ribosomal subunit. The *dys1-1* mutant was synthetically lethal in combination with *asc1Δ* and overexpression of *TIF51A* (eIF5A) or *DYS1* is toxic for an *asc1Δ* strain. Moreover, eIF5A is more associated with translating ribosomes in the absence of Asc1 in the cell. Finally, analysis of the sensitivity to cell wall-perturbing compounds revealed a more similar behavior of the *dys1-1* and *asc1Δ* mutants in comparison with the *pkc1Δ* mutant. These data suggest a correlated role for eIF5A and Asc1 in coordinating the translational control of a subset of mRNAs associated with cell integrity.

## Introduction

Initially purified from the ribosomes of reticulocyte lysates, the putative eukaryotic translation initiation factor 5A (eIF5A) was shown to stimulate the synthesis of methionyl-puromycin *in vitro*, indicating a role in the formation of the first peptide bond [1]. However, the depletion of eIF5A in yeast only showed a small decrease in the overall rate of protein synthesis, arguing against an essential role for eIF5A in general translation [2], [3]. Therefore, it has been proposed that eIF5A plays a role in the translation of mRNAs encoding specific proteins, such as those required for cell cycle progression [4].

eIF5A has also been implicated in several mechanisms, such as mRNA decay. However, the time of growth arrest of the yeast eIF5A mutants used in the studies do not correlate with the time of mRNA degradation defect [5], the mRNA degradation defect and block of formation of P-bodies is identical to the efect of the translation elongation blocker cycloheximide [6] and another translation elongation mutant, the *cca1-1*, also demonstrate an mRNA degradation defect [7], suggesting that the effect of eIF5A on mRNA decay is actually secondary. Besides, eIF5A was involved with the nuclear export of HIV1 Rev by the Crm1/Xpo1 transporter [8]–[10], nevertheless, results generated by other groups failed to confirm physical and functional interactions between eIF5A and HIV-1 Rev or the Crm1/Xpo1 transporter in different experimental models [5], [11]–[14].

Although there might be a role for eIF5A in translation initiation [15], it has been demonstrated that eIF5A physically interacts with the 80S ribosome and translation elongation factors [16], [17] and functionally interacts with elongation factor 2 [18]. Furthermore, the accumulation of polysomes and an increase in the average ribosomal transit time has been observed in yeast eIF5A mutants, supporting a role for eIF5A in translation elongation instead of translation initiation [6], [19].

Interestingly, eIF5A requires a unique and essential posttranslational modification in which a specific lysine is converted to hypusine. This specific lysine (K51 in *S. cerevisiae*) is located at the tip of an exposed loop in the N-terminal domain of eIF5A [20]–[23]. eIF5A is the only protein containing a hypusine residue, which results from a posttranslational modification in which the enzyme deoxyhypusine synthase (Dys1 in *S. cerevisiae*) transfers an aminobutyl moiety from polyamine spermidine to the amino group of a specific lysine residue to form deoxyhypusine, followed by the addition of a hydroxyl group, which is catalyzed through deoxyhypusine hydroxylase (Lia1 in *S. cerevisiae*) activity [4]. Curiously, the deoxyhypusine synthase gene (*DYS1*) is only essential for growth in *S. cerevisiae*, and deoxyhypusine hydroxylase function is only essential in higher eukaryotes [24], [25]. Although posttranslational modification is essential for elF5A activity, and the mechanism of hypusination has been extensively characterized, the role of the hypusine residue in eIF5A remains obscure.

To further investigate the function of eIF5A and its unique hypusine residue, we generated a conditional *DYS1* mutant at the *dys1-1* allele and characterized its hypusine content, growth phenotype, total protein synthesis and polysome profile. We also identified the genetic interactions of the *dys1-1* mutant with *PKC1* and *ASC1* mutants, implicating a role for eIF5A and Asc1 in the maintenance of cell integrity at the translational level in a distinct pathway associated with the well-known Pkc1 pathway.

## Results

### The Osmotic Stabilizer Requirement for *dys1-1* Growth is not Associated with Cell Lysis

**Figure 1:**
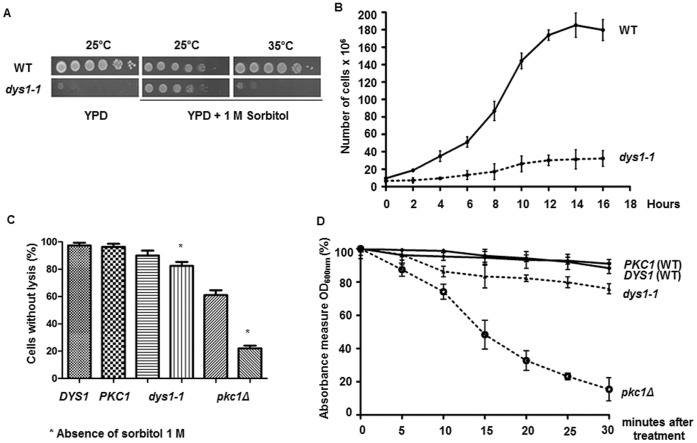
The *dys1-1* mutant shows a severe growth defect that is not associated with cell lysis. (A) Serial dilutions of the wild type and *dys1-1* mutant strains were plated onto YPD medium in the presence or absence of 1 M sorbitol at the indicated temperatures. (B) Growth curves of the wild type and *dys1-1* mutant strains. The strains were grown at 25°C in YPD medium containing 1 M sorbitol, and the cell numbers were counted to monitor the 16- h growth rate. (C) The cells were grown as in Figure 1B, to mid-log phase, treated with methylene blue and counted to analyze the cell lysis. The quantification is shown relative to the wild type (100%). (D) Sensitivity to zymolyase. The cells were grown as in Figure 1C and treated with zymolyase. The turbidity was measured at the indicated time points after the cells were treated with SDS.

To generate a conditional *DYS1* mutant for the characterization of the role of the hypusine modification in eIF5A, we used site-directed mutagenesis to target conserved residues of deoxyhypusine synthase and selected for mutations that would impair the capacity of the *DYS1* gene to complement a *dys1Δ* yeast strain. Because it has previously been shown that eIF5A mutants exhibit suppression of the temperature-sensitive phenotype in the presence of an osmotic stabilizer such as sorbitol [5], [26], we supplemented the growth medium with 1 M sorbitol. One of the mutants generated was able to complement the *dys1Δ* yeast strain in the presence of an osmotic stabilizer at 25°C (permissive condition) and also showed a temperature-sensitive phenotype at 35°C (restrictive condition) (Figure 1A). Furthermore, this mutant exhibited a reduced growth rate, even under the permissive growth condition (Figure 1B). These phenotypes demonstrate a more severe growth defect when compared with eIF5A mutants [5], [26].

As expected from the mutation of conserved residues, this mutant carried a T118A substitution in the Dys1 protein. However, two additional and unexpected mutations (W75R and A147T) were revealed in this mutant. When verified, W303 background strains naturally contained R75 and T147 instead of W75 and A147, which are found in S288C background strains. We also generated the T118A mutation in the S288C *DYS1*, but no phenotype was observed. Moreover, a *dys1Δ* yeast strain complemented by either W303 *DYS1* (R75, T147) or S288C *DYS1* (W75, A147) alleles show no distinguishable phenotypes (data not shown). Therefore, although R75 and T147 are naturally occurring variations in the sequence coded by *DYS1* in W303 and do not impair Dys1 function in this background, together with the T118A mutation, they significantly impair Dys1 function. The triple mutant *dys1* ^W75R, T118A, A147T^ was named here *dys1-1*.

Mutations in the Pkc1 cell integrity pathway result in an osmolarity support for vegetative growth phenotype due to increased cell lysis [27]. To determine whether the *dys1-1* mutant shows increased lysis compared with the wild type strain, we conducted methylene blue staining and zymolyase sensitivity assays according to standard procedures [28], [29]. As observed in the vital staining experiment (Figure 1C), the *dys1-1* mutant revealed almost no cell lysis in the presence of 1 M sorbitol and a small cell lysis defect (<20%) in the absence of 1 M sorbitol, a condition in which the *dys1-1* mutant does not grow (Figure 1A). A similar result was observed in the experiment using zymolyase (Figure 1D). However, the *pkc1Δ* mutant showed almost 40% cell lysis, even in the presence of 1 M sorbitol, and approximately 80% cell lysis in the absence of 1 M sorbitol. These results suggest a minor cell lysis defect in the *dys1-1* mutant, which could not produce the severe growth impairment observed for this mutant. Therefore, although the *dys1-1* mutant is viable only in the presence of an osmotic stabilizer (1 M sorbitol), this phenotype does not reflect cell lysis, as shown for cell wall integrity mutants, such as *pkc1Δ* [27].

### The *dys1-1* Mutant is Hypusine Deficient and shows a Defect in Translation Elongation

**Figure 2:**
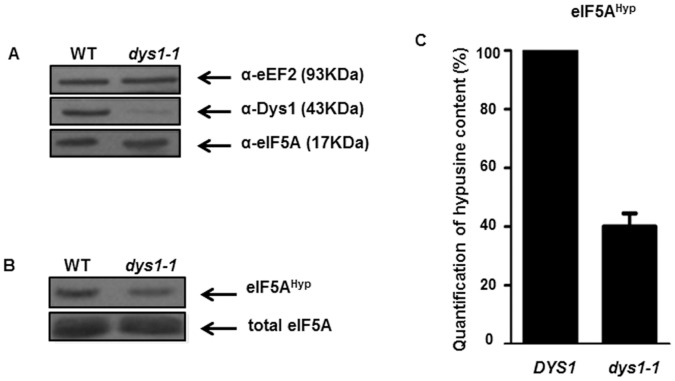
The *dys1-1* mutant reveals a drastic reduction in Dys1 protein levels resulting in a reduction in hypusine-containing eIF5A. (A) Determination of mutant Dys1 protein levels. Wild type and *dys1-1* mutant strains were grown to mid-log phase at the permissive temperature in YPD medium containing 1 M sorbitol. The cells were lysed and 10 µg of total protein were blotted with the indicated antibodies. Samples were probed for eEF2 as a loading control. (B) Detection of hypusine-containing eIF5A of wild type and *dys1-1* mutant strains. Total eIF5A was immunoprecipitated and subjected to SDS-PAGE. Hypusine-containing eIF5A was revealed by autoradiography. (C) Quantification of relative hypusination levels after analysis of hypusine-containing versus total eIF5A, comparing the *dys1-1* mutant with the wild type (*DYS1*) and expressed the quantification as percent of wild type.

To further characterize the *dys1-1* mutant, we first analyzed the expression of Dys1 and hypusine-containing eIF5A protein. The haploid yeast strain, carrying only the *dys1-1* allele, showed a dramatic decrease in Dys1 protein levels and an expected reduction in the amount of hypusine-containing, but not total, eIF5A (Figure 2A and 2B). The quantification of the hypusine-containing eIF5A protein revealed that the *dys1-1* mutant showed a 60% decrease compared with wild type cells (Figure 2C).

**Figure 3:**
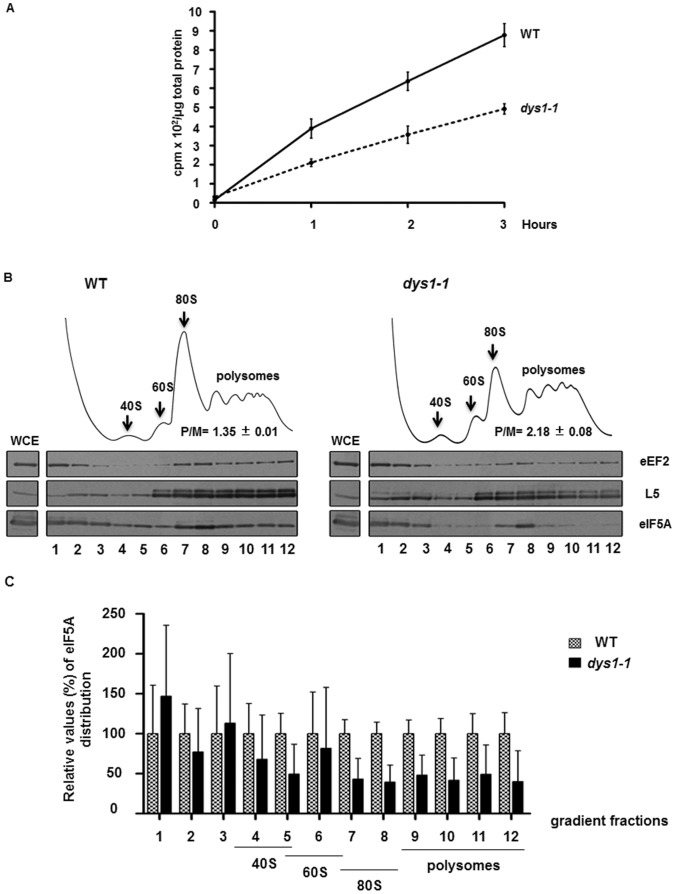
The reduction in hypusine formation in *dys1-1* mutant results in a reduction in total protein synthesis, and the polysome profile is characteristic of translation elongation defects. (A) The indicated strains were grown to mid-log phase, as in Figure 1B and radiolabeled [^3^H]leucine was added to the medium. The incorporation of [^3^H]leucine into total proteins was measured as described in the Materials and Methods. (B) Whole cell extracts (WCE) of the indicated strains were fractionated through centrifugation in a sucrose density gradient. Optical scans (OD_254nm_) of the gradients are shown. The areas of the 80S and polysome peaks were compared to calculate the P/M ratio. The polysome profile fractions and the WCE were collected and blotted against the indicated antibodies. (C) Quantification of the ribosome-bound eIF5A relative to the amount of ribosomes (normalized by ribosomal protein L5) in the polysome profile fractions. The values obtained with the wild type strain were considered as 100% and those obtained with mutant strains were expressed as percentages of the wild type in the bar graphs.

As previously demonstrated, different eIF5A mutants display a significant decrease in total cellular protein synthesis, and the polysome profiles showed an increase of polysomes compared with monosomes, consistent with a defect in translation elongation [6], [19]. As the modification hypusine is essential for eIF5A activity [24], we examined whether the *dys1-1* mutant strain would also show these defects. As observed in Figure 3A, protein synthesis analysis of the *dys1-1* mutant revealed a 50% decrease in [^3^H]leucine incorporation in total cellular protein. Moreover, the polysome profile analysis of the *dys1-1* mutant demonstrated a significant increase in the polysome to monosome (P/M) ratio (Figure 3B, upper panels; Figure S4). These results are consistent with defects in translation elongation and support the idea that eIF5A plays a role in this step of protein synthesis. These findings reveal that the reduced levels of hypusine formation in the *dys1-1* mutant results in translation elongation defects similar to those observed for eIF5A mutants [6], [17], [19], [26].

We also investigated the association of eIF5A with ribosomes purified from the fractionation of the polysome profiles of wild type and *dys1-1* mutant strains. Although no decrease in total eIF5A levels was observed in the *dys1-1* mutant (Figure 2B), a significantly reduced amount of eIF5A was associated with polysomal fractions in this mutant (Figure 3B and 3C). This observation is consistent with our previous data showing that the mutant eIF5A^K51R^, which is defective for hypusine modification, is significantly impaired for ribosome binding [17].

### *DYS1* and *TIF51A* Genetically Interacts with *ASC1* and eIF5A Binding to Translating Ribosomes is Enhanced in the Absence of Asc1

**Figure 4:**
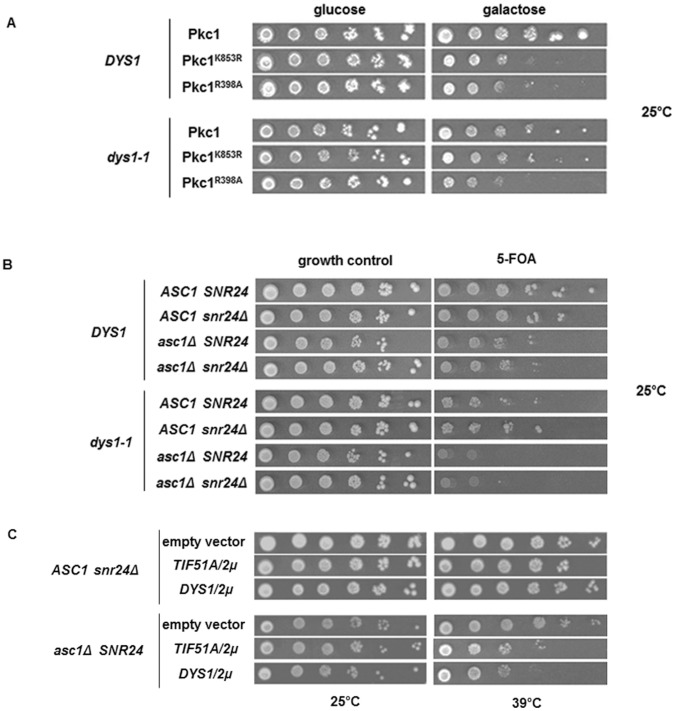
Dys1 and eIF5A genetically interact with Pkc1 and Asc1. (A) The wild type and mutant strains harboring wild type, inactive or constitutively active forms of Pkc1 protein under a galactose-inducible promoter were plated onto SC-ura medium containing 1 M sorbitol plus glucose (growth control) and plus galactose (inducible condition) and grown at 25°C for 3 days. (B) The indicated strains were plated onto medium not containing or containing 5-FOA and grown at 25°C for 3 days for plasmid shuffle. (C) The indicated strains harboring the empty vector, *TIF51A* or *DYS1* high-copy plasmids (*2μ*) were grown at the permissive and restrictive conditions for 3 days.

The overexpression of *PKC1* suppresses the temperature-sensitive phenotype of the *tif51A-1* mutant of eIF5A [5]; therefore, we determined whether the overexpression of *PKC1* also rescues the growth defects of the *dys1-1* mutant. However, neither the requirement for osmolarity support nor the temperature sensitivity of the *dys1-1* mutant was suppressed after the overexpression of *PKC1* (data not shown). We also determined whether the overexpression of inactive (K853R) or constitutively active (R398A) Pkc1 affects the growth of the *dys1-1* mutant. Interestingly, whereas both mutated forms of Pkc1 reduced the growth of the wild type strain (Figure 4A, upper panels), the inactive Pkc1 (K853R) form did not affect the *dys1-1* mutant (Figure 4A, lower panels).

Curiously, the *asc1Δ* mutant also exhibits a marked resistance to the overexpression of the inactive Pkc1 mutant (K853R) [29]. Asc1 is an ortholog of mammalian RACK1, a core component of the small (40S) ribosomal subunit that is also involved in Gpa2 signaling in response to glucose sensing as a G-protein β-subunit [30], [31]. Because both eIF5A and Asc1 are directly associated with translation and genetically related to *PKC1*, we characterized the genetic interaction between eIF5A mutants or the *dys1-1* mutant with the *asc1Δ* mutant.

Notably, an intron of *ASC1* contains the gene *SNR24* encoding for the small nucleolar RNA (snoRNA) U24 [32], which is involved in the maturation of the large subunit rRNA [33]. To distinguish the genetic interactions with *ASC1* from those with *SNR24*, the genetic analysis was performed in a double *asc1Δ snr24Δ* haploid mutant strain complemented with *ASC1* (without *SNR24*), *SNR24* alone or the entire *ASC1* gene (*ASC1*+*SNR24*) (Figure S1).

Although a clear synthetic lethality between the eIF5A mutants and the *asc1Δ* mutant was not observed (data not shown), the haploid strain harboring both *dys1-1* and *asc1Δ* alleles was not viable, suggesting synthetic lethality between these two genes (Figure 4B). This genetic interaction was specific to the absence of *ASC1* alone, as the strain without the *SNR24* gene did not exhibit a synthetic lethality with the *dys1-1* mutant. This synthetic lethality between *dys1-1* and *asc1Δ* suggests that wild type levels of hypusine-containing eIF5A in the cell are necessary to compensate for the absence of Asc1 function, and therefore both proteins might function at the translational level to ensure the correct expression of the genes associated with cell wall integrity.

We further examined whether the overexpression of *ASC1* was able to suppress the conditional growth phenotypes of the eIF5A and *dys1-1* mutants. However, no suppression was observed (Figure S2). We also examined the effect of eIF5A (*TIF51A*) and *DYS1* overexpression in the *asc1Δ* mutant. As shown in Figure 4C, the overexpression of both *TIF51A* or *DYS1* was toxic in the *asc1Δ* mutant (lower panels), but had no effect on the growth of the wild type *ASC1* strain (upper panels). This toxic effect was reproducible and suggests that eIF5A and Asc1 act in a competitive manner to differentially regulate mRNA translation in the cell.

**Figure 5:**
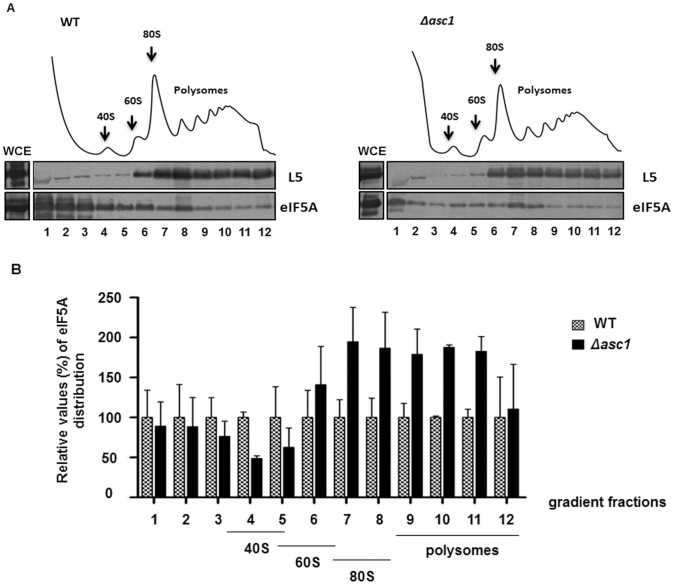
Absence of Asc1 increases association of eIF5A to polysome profile fractions. (A) Whole cell extracts (WCE) of the indicated strains were fractionated through centrifugation in a sucrose density gradient. Optical scans (OD_254nm_) of the gradients are shown. The polysome profile fractions and the WCE were collected and blotted against the indicated antibodies. (B) Quantification of the ribosome-bound eIF5A relative to the amount of ribosomes (normalized by ribosomal protein L5) in the polysome profile fractions. The values obtained with the wild type strain were considered as 100% and those obtained with mutant strains were expressed as percentages of the wild type in the bar graphs.

We also tested whether presence or absence of Asc1 in the cell influences eIF5A binding to ribosomes. As shown in Figure 5, quantification of eIF5A binding to ribosomes, relative to ribosomal protein L5 as a loading control, demonstrates an increase of eIF5A association with 80S and polysome fractions in the absence of Asc1. The increase in eIF5A binding to translating ribosomes is in agreement with the idea that there must be a balance between Asc1 and eIF5A in the control of protein synthesis.

**Figure 6:**
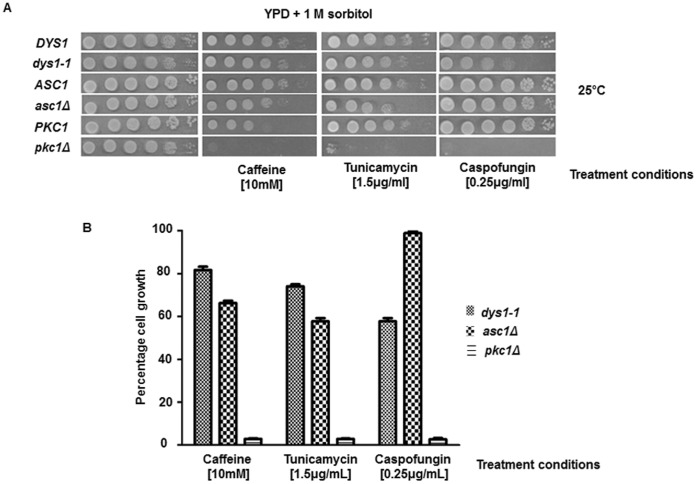
The Dys1, Asc1 and Pkc1 mutants showed a distinguished sensitivity to compounds affecting cytoplasmic membrane and cell wall integrity. (A) The strains were plated onto medium supplemented with the indicated drugs and grown at 25°C for 3 days. (B) The growth was measured relative to each respective isogenic wild type strain (100%).

In addition, to evaluate the biological effects of Asc1 and eIF5A/Dys1 in the cell, we examined the sensitivity of *asc1*Δ and *dys1-1* mutants to three compounds that affect cytoplasmic membrane and/or cell wall integrity: caffeine, a phosphodiesterase inhibitor that activates the Pkc1p-MAP kinase cell integrity pathway; tunicamycin, a general inhibitor of protein N-glycosylation; and caspofungin, an inhibitor of β(1,3)-glucan synthase. The different genetic strain backgrounds significantly affected the sensitivity to some compounds, and the sensitivity analysis using the *tif51A-1* mutant of eIF5A was not informative (data not shown). However, comparing *dys1-1*, *asc1Δ* and *pkc1Δ* with their respective wild type controls revealed that the *dys1-1* and the *asc1Δ* mutants were much less sensitive to all three compounds than the *pkc1Δ* mutant (Figure 6A and 6B). The *asc1Δ* mutant did not show any sensitivity to caspofungin.

These results demonstrate a broader sensitivity of the *pkc1Δ* mutant compared with the *dys1-1* and *asc1Δ* mutants, potentially reflecting a more direct role for Pkc1 in several pathways involved in cell wall maintenance, while Asc1 and the hypusine-containing eIF5A protein might impact the cell membrane and cell wall integrity in a more specific manner through the control of gene expression at the translational level.

### Genetic Interaction between *DYS1* and *ASC1* is Dependent on Asc1 Binding to the Ribosome

The association of Asc1 with the 40S ribosomal subunit has been well established, and its association with the ribosome is required for translation-associated functions [34]–[36]. That is, the absence of Asc1 in the cell causes phenotypes associated with the process of protein synthesis, and *ASC1* point mutations generate Asc1 protein with reduced ribosome binding and also exhibit these phenotypes [34], [35]. However, ribosome binding is not required for Asc1 function as a G-protein β-subunit in glucose sensing, as the mutant *asc1^R38D,K40E^*, which shows reduced binding to the 40S subunit, is not defective in haploid invasive growth [34].

**Figure 7:**
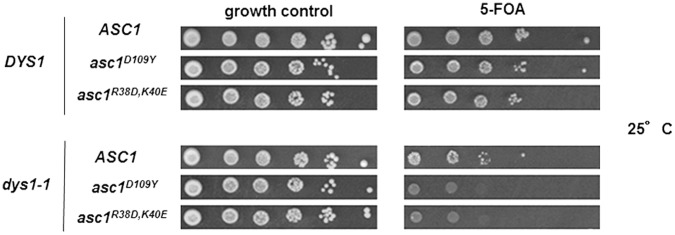
Genetic interaction between Dys1 and Asc1 is related to Asc1 binding to the 40S ribosome subunit. The indicated strains were plated onto medium not containing or containing 5-FOA and grown at 25°C for 3 days for plasmid shuffle.

To confirm that the functional link between hypusine-containing eIF5A and Asc1 is associated with translation, we characterized the genetic interaction between the *dys1-1* mutant and the mutants *asc1^R38D,K40E^* and *asc1^D109Y^*, which generate Asc1 proteins defective in binding to the 40S ribosomal subunit [34], [35]. As shown in Figure 7, *dys1-1* was synthetically lethal with both *asc1^R38D,K40E^* and *asc1^D109Y^* mutants, phenocopying the data of synthetic lethality observed between *dys1-1* and *asc1Δ* (Figure 4B). Unfortunately, there is no *ASC1* point mutant that specifically shows defects in the glucose-sensing pathway.

This result suggests that the functions of Asc1 associated with protein synthesis are necessary in the absence of wild type levels of hypusine-containing eIF5A in the cell and further supports the idea that functions of Asc1 and eIF5A in mRNAs the translation are required for cell viability.

## Discussion

The functional characterization of the putative translation factor eIF5A has primarily been conducted in the model organism *S. cerevisiae* [37], [38]. Although several conditional mutants of eIF5A have previously been isolated and used in different studies, no conditional mutant for the gene encoding the enzyme Dys1, which is responsible for deoxyhypusine formation in eIF5A, has been described so far. As hypusine modification is essential for the function and ribosome binding of elF5A, the use of a conditional mutant of *DYS1* might provide insight into the aspects specifically associated with the loss of the hypusine residue in eIF5A, instead of using the depletion of eIF5A in the cell. Herein, we describe the generation of a conditional *dys1-1* mutant to further characterize the role of eIF5A and the hypusine residue in the maintenance of cell integrity.

The *dys1-1* mutant shows a marked reduction of Dys1 protein levels and a consequent reduction of hypusine-containing eIF5A. Therefore, *dys1-1* is an interesting mutant to study the function of the hypusine residue in eIF5A and compare the effects of hypusine depletion versus total eIF5A depletion in the cell. Although eIF5A is an abundant protein, our data suggest that hypusine content less than half of that of wild type (∼40%) in the *dys1-1* mutant is not sufficient to promote protein synthesis optimally and, therefore, results in severe growth defects. On the other hand, it could be suggested that non-hypusinated eIF5A has a deleterious effect on protein synthesis. However, the non-hypusinatable eIF5A^K51R^ protein does not bind to the translational machinery or any other proteins in general [17], [39] and, hence, could not compete with hypusine-containing eIF5A. Also, overexpression of the eIF5A^K51R^ mutant had no deleterious effect on growth of a wild type yeast strain (Figure S3). In addition, the conditional mutant eIF5A^K56A^, which shows a reduced content of hypusinated eIF5A to approximately 60% of that of the wild type, also shows a growth defect already at the permissive temperature [26].

In addition to exhibiting defects associated with protein synthesis and translation machinery, the *dys1-1* mutant showed complete dependence on an osmotic stabilizer (1 M sorbitol) for growth, which is a well-known phenotype for mutants of the Pkc1 cell wall integrity pathway, such as the *pkc1Δ* mutant [27]. A similar phenotype has also been observed for temperature-sensitive eIF5A mutants, which are suppressed using an osmotic stabilizer at the restrictive temperature [5], [26]. Nevertheless, while the *pkc1Δ* mutant requires an osmotic stabilizer due to lysis of a large fraction of cells in liquid culture (approximately 80%) and exhibits hypersensitivity to zymolyase treatment, the *dys1-1* mutant shows only a modest amount of cell lysis in the absence of osmotic stabilizer (less than 20%) and a minor sensitivity to zymolyase treatment compared with the wild type control. These results suggest that cell lysis does not influence the severe growth defect phenotype of the *dys1-1* mutant, even in the presence of the osmotic stabilizer. Therefore, we suggest that, although eIF5A is functionally associated with Pkc1 signaling [5], the consequence of depleting the hypusine modification in eIF5A does not completely mimic the lack of Pkc1 function in the cell.

We also show that the *dys1-1* mutant is resistant to the toxic effects of the overexpression of the inactive Pkc1^K853R^ mutant, which is consistent with a previous result obtained for the *tif51A-1* mutant [5]. This genetic interaction further supports the hypothesis that eIF5A and Pkc1 function in different cellular pathways to maintain cell integrity. Because the *asc1Δ* mutant also showed resistance to the toxic effects of the overexpression of the inactive Pkc1^K853R^ mutant [29], we analyzed the functional correlation between eIF5A, Dys1 and Asc1. Interestingly, both eIF5A and Asc1 have previously been associated with the translation of specific mRNAs necessary for correct bud formation during cell cycle progression or bud site localization, respectively [29], [40].

The synthetic genetic interaction revealed between the *dys1-1* and *asc1Δ* mutants suggest that these factors are functionally linked. Moreover, the overexpression of either *TIF51A* or *DYS1* causes cell toxicity in the absence of *ASC1* and eIF5A binding to the translating ribosomes is enhanced in the absence of Asc1. Taken together, these genetic interactions suggest that a precise balance between hypusine-containing eIF5A and Asc1 is necessary for the correct expression of cell integrity-related genes at the translational level. In addition, *dys1-1* and *asc1Δ* mutants tend to show a similar pattern of sensitivity to compounds that interfere with cell integrity and are less sensitive than the *pkc1Δ* mutant. These results further support a closer functional link between eIF5A and Asc1 compared with Pkc1. Furthermore, the genetic interaction between eIF5A/Dys1 and Asc1 is associated with the ability of Asc1 to bind to the 40S ribosomal subunit, confirming that these proteins are associated through their respective functions in translation.

The increased eIF5A binding to translating ribosomes in the absence of Asc1 may favor translation of a group of mRNAs different from those mRNAs recently demonstrated to be regulated by Asc1 [41]. The precise mechanism whereby eIF5A can differentially affect translation of specific mRNAs and which mRNAs are more dramatically influenced by eIF5A function in translation are currently being addressed in our laboratory and further efforts will be necessary to address these questions. Interestingly, the structural homologue of eIF5A in eubacteria, EF-P, has recently been demonstrated to affect membrane integrity, cell response to enviromental stresses, virulence and motility [42]–[47]. Also, EF-P more drastically affects the translation of poly-proline tract-containing proteins [48], [49]. However, the precise mRNAs that are translated less in the absence of EF-P to lead to the observed phenotypes are not yet determined. Whether eIF5A also affects translation of proteins harboring consecutive proline residues is also under investigation.

Finally, Asc1 has been implicated in the maintenance of cell wall integrity near bud sites [29], which likely occurs after bud emergence during the G1 phase of the cell cycle. Hypusine-containing eIF5A has also been implicated in cell cycle progression during G1 [40], [50] for the positive control of the expression of cell integrity mRNAs, which might also occur in a cell cycle-related manner. The suggestion that eIF5A is more associated with Asc1 than to Pkc1 could explain the discrete cell lysis phenotype observed during the depletion of hypusine modification in eIF5A in the *dys1-1* mutant, and because Pkc1 is important for cell wall integrity during the entire cell cycle, Pkc1 mutants display a major cell lysis defect in the absence of an osmotic stabilizer.

## Materials and Methods

### Yeast Strains, Plasmids and Standard Procedures

The yeast strains are listed in Table 1. The plasmids used in this study include pRS314 (*TRP1/CEN*), pRS316 (*URA3/CEN*), pRS426 (*URA3/2μ*), pRS424 (*TRP1/2μ*), pSV223 (Gal1 promoter - *PKC1/URA3/2µ*), pSV224 (Gal1 promoter - *PKC1^K853R^/URA3/2µ*), pSV225 (Gal1 promoter - *PKC1^R398A^/URA3/2µ*). The procedures for cell growth and genetic manipulations were performed according to standard protocols [51].

**Table 1:** Yeast strains used in this study.

Strain	Genotype	Source
SVL82 (W3O3)	*MATa ade2-1 his3-11,15 leu2-3,112 trp1-1 ura3-1 can1-100 ssd1-d*	Pamela Silver
SVL95	*MATa leu2 trp1 ura3 his4 can1 pkc1::LEU2*	Anne McBride
SVL132	*MAT*α *leu2 ura3 his3 tif51A::HIS3* [*TIF51A/URA3/CEN* - pSV138]	Lab Collection
VZL272 (BY4741)	*MAT* a *leu2 ura3 his3 met15*	Anita Corbett
SVL452	*MAT* a *leu2 trp1 ura3 his3 dys1::HIS3* [*DYS1/URA3/CEN -* pSV526]	This study
SVL453	*MAT*α *leu2 trp1 ura3 his3 dys1::HIS3* [*DYS1/URA3/CEN -* pSV526]	This study
SVL613	*MATa leu2 trp1 ura3 his3 dys1::HIS3* [*DYS1/TRP1/CEN* - pSV520]	This study
SVL 614	*MATa leu2 trp1 ura3 his3 dys1::HIS3* [*dys1* ^W75R,T118A,A147T^ */TRP1/CEN* - pSV730]	This study
VZL1133	*MAT* a *ura3 his3 leu2 met15 asc1::kanMX4*	Knockout Collection
VZL1134	*MAT*α *ura3 his3 leu2 trp1 met15 asc1::kanMX4 dys1::HIS3* [*DYS1/URA3/CEN -* pSV526]	This study
VZL1173 (EG123)	*MATa leu2 trp1 ura3 his4 can1*	Arava Yoav
VZL1178	*MAT*α *ura3 his3 trp1 leu2 met15 asc1::kanMX4 tif51A::HIS3* [*TIF51A/URA3/CEN -* pSV138]	This study

### Isolation of Conditional *DYS1* Mutants

The *DYS1* mutants were generated using site-directed mutagenesis in which a plasmid vector (pRS314) containing *DYS1* from the W3O3 wild type strain was used as a template. To generate the mutations, site-directed mutagenesis was performed on selected conserved residues in Dys1 using the QuickChange site-directed mutagenesis kit (Stratagene, La Jolla, CA, USA) according to the manufacturer’s instructions. The presence of the specific mutations was confirmed through DNA sequencing. The plasmid constructs containing different mutated *DYS1* alleles were introduced into the SVL452 strain and plated onto SC media supplemented with 5-fluoroorotic acid (5-FOA) to negatively select cells that did not contain the *URA3* plasmid (plasmid shuffle). The resulting Dys1 mutant strains, which complemented the absence of wild type allele, did not exhibit a temperature-sensitive phenotype. Subsequently, the mutated alleles that did not complement the *dys1Δ* strain were reintroduced into the SVL452 strain and plasmid shuffle was performed in the presence of an osmotic stabilizer (1 M sorbitol), as sorbitol suppresses the thermosensitivity of eIF5A mutants [5].

### Western Blot Analysis

To determine specific protein levels in the strains used in this study, the cells were grown to mid-log phase under permissive conditions and subsequently lysed in protein extraction buffer (20 mM Tris/HCl, pH 7.5; 2 mM dithiothreitol; 2 mM EDTA and 5 µg.mL^-1^ of pepstatin, leupeptin, aprotinin and chymostatin). Total protein was resolved using SDS-PAGE and transferred to nitrocellulose. The proteins of interest were detected through immunoblotting with specific antibodies using a chemiluminescence detection system.

### *In vivo* Hypusine Synthesis

Yeast strains carrying the wild type or *dys1-1* allele were grown to mid-log phase (OD_600nm_ = 0.5) at 25°C in 5 mL of YPD medium. The cultures were diluted and [^3^H]spermidine (PerkinElmer, MA, USA) was added to the medium to a final concentration of 7 µCi.mL^-1^. The cultures were subsequently incubated at 25°C, grown to the log phase (OD_600nm_ = 1.0), harvested at 4°C and frozen at −80°C. The yeast extracts containing radiolabeled eIF5A were subjected to eIF5A immunoprecipitation, as previously described [52]. The cell pellet was resuspended and subjected to SDS-PAGE analysis. The polyacrylamide gel was stained with Coomassie Blue and total eIF5A was observed. To analyze the levels hypusine-containing eIF5A, the polyacrylamide gel was incubated with Amplify (Amersham Bioscience), dried and exposed to an autoradiography film. The quantification was performed using the Image Scanner III and Image Quant TL software (GE Healthcare Life Sciences) and the data were expressed as the percentage relative to the wild type (100%).

### Protein Synthesis Assay

Yeast strains carrying the wild type or *dys1-1* allele were grown to mid-log phase (OD_600nm_ = 0.5) at 25°C in 10 mL of YPD medium. [^3^H]leucine (PerkinElmer, MA, USA) was added to the medium to a final concentration of 2 µCi.mL^-1^. The cultures were subsequently incubated at 25°C, and 1.5 mL aliquots from the cultures were collected at 1, 2 and 3 h after the addition of [^3^H]leucine, harvested at 4°C and frozen at −80°C. All frozen cell pellets were resuspended in 15% cold trichloroacetic acid solution and incubated on ice for 15 min. The samples were heated at 72°C for 30 min and subsequently incubated on ice for 15 min. Trichloroacetic acid precipitates were collected using centrifugation (15,000 *g*, 4°C, 10 min) and washed four times with 10% trichloroacetic acid to remove free [^3^H]leucine. The final washed pellets were resuspended in 100 µL of 0.2 M NaOH, and 50 µL aliquots were used to determine the radioactivity incorporated during protein synthesis in a Beckman Scintillation Counter (Beckman Coulter, Brea, CA, USA). The total protein concentration was determined using the BCA protein assay (Thermo Scientific, IL, USA), with 5 µL aliquots of each sample. The amount of total protein synthesis was calculated for each sample as c.p.m.µg^-1^ total cell protein.

### Polysome Profiling using Formaldehyde

The cells from the 100 mL cultures were grown to mid-log phase (OD_600nm_ = 0.6) and cross-linked with 1% formaldehyde for 1 h in an ice bath. The extracts were used for each sucrose gradient. Briefly, 15 A_260nm_ units of cell lysates were layered onto 10–50% sucrose gradients and centrifuged for 3 h at 39,000 rpm at 4°C in a Beckman SW41-Ti rotor. The gradients were subsequently fractionated through upward displacement with 60% (w/v) sucrose using a gradient fractionator connected to a Control Unit UV-1 monitor (Amersham Pharmacia Biotech) for the continuous measurement of the absorbance at 254 nm. The polysomal profile fractions were quantified using NIH Image J software. The polysome profile sucrose gradient fractions were collected, and the proteins were precipitated with acetone and subjected to western blot analysis. Quantification of eIF5A associated to ribosomes was performed as before [36]. Briefly, western blot signals of all the fractions were quantified using ImageScanner III (GE Healthcare, Life Sciences) and normalized to 60S ribosomal protein L5 levels, and the mean eIF5A/60S ratio was determined from at least three replicate experiments. The values obtained for the indicated yeast strains were then plotted in percentages relative to wild type.

### Growth Analysis

#### Solid medium

The strains of interest were grown at the permissive condition and equal amounts of the cultures were subsequently harvested. Ten-fold serial dilutions were plated onto specific media (supplemented or not with different compounds) and grown at the indicated temperatures for 3 or 4 days. **Liquid medium (growth curve).** The strains of interest were grown at the permissive condition to mid-log phase and subsequently diluted 10^7^ cells.mL^-1^. At this time, the cells were counted to monitor the time course of growth.

### Synthetic Lethality

The double knockouts were generated by standard crossing and sporulation methods. Spores of interest were selected in the presence of geneticin (*asc1Δ::kanMX4*) and absence of histidine and uracil (*dys1Δ*::*HIS3* harboring *DYS1*/*URA3*/*CEN*). The strains were then transformed with *dys1-1*/*TRP1*/*CEN* or *DYS1/TRP1/CEN* plasmids and with other plasmids containing the genes indicated in the respective figures. After transformation, a plasmid shuffle [53], allowed exchange of the *DYS1*/*URA3*/*CEN* for the *dys1-1*/*TRP1*/*CEN* or *DYS1/TRP1/CEN* plasmids. Briefly, the plasmid shuffle assay was used to select the strains that, due to natural plasmid loss, do not harbor the *URA3* plasmid after growth in media containing uracil. The selection against the *URA3* plasmid is due to the toxic effect of 5-fluoro-orotic acid (5-FOA) in cells that harbor a *URA3* gene, since the 5-FOA is converted into a highly toxic metabolite (5-fluorouracil) by the *URA3* gene product.

### Drug Sensitivity Assay

The indicated strains were grown and manipulated as described in Growth analysis for solid medium, plated in the presence of the indicated compounds and grown at 25°C for 3 days. The quantification of cell growth was performed by visual analysis of the plates, considering the growth of the strains in the serially diluted spots, normalized to their respective isogenic wild type strains, assuming growth of wild type strains as 100%. All the growth analyses were performed with 3 different colonies of each strain and repeated 3 times.

## Supporting Information

Figure S1 **The** ***dys1-1*** **mutant grows only in the presence of** ***ASC1*** **.** The indicated strains harboring *DYS1*, *dys1-1* or empty vector were transformed with *ASC1* (without *SNR24*), *SNR*24 alone, the entire *ASC1* gene (*ASC1*+ *SNR24*) or the empty vector and plated onto medium not containing or containing 5-FOA and grown at 25°C for 3 days for plasmid shuffle.
(TIF)

Figure S2 **High-copy** ***ASC1*** **did not suppress the** ***dys1-1*** **and** ***tif51A-1*** **growth defects.** The *dys1-1* and *tif51A-1* mutants harboring *ASC1* in high-copy plasmid (*2μ*) were grown at permissive and restrictive conditions for 3 days.
(TIF)

Figure S3 **Overexpression of eIF5A^K51R^ does not affect growth of wild type cells.** Serial dilutions of wild type SVL272 transformed with vector pYES2, pSV975 (pYES2-*TIF51A*) and pSV976 (pYES2-*tif51A^K51R^*) were plated onto SC-ura supplemented with 2% glucose (growth control) or galactose (to induce heterologous eIF5A expression) and incubated at permissive temperature for 2 days.
(TIF)

Figure S4 **Polysome profiling of** ***dys1-1*** **mutant after treatment with cycloheximide reveals the same defect found following formaldehyde crosslinking.** Whole cell extracts (WCE) of the indicated strains, after treatment with cycloheximide, were fractionated through centrifugation in a sucrose density gradient. Optical scans (OD_254nm_) of the gradients are shown. The areas of the 80S and polysome peaks were compared to calculate the P/M ratio.
(TIF)

Method S1 **Polysome profiling using cycloheximide.** The cells from 200-mL cultures were grown to mid-log phase and treated with 10 µg/mL of cycloheximide for 5 min. The extracts were used for each sucrose gradient. Briefly, 20 A_260nm_ units of cell lysates were layered onto 7–47% sucrose gradients containing 10 µg/mL of cycloheximide and centrifuged for 3 h at 39,000 rpm at 4°C in a Beckman SW41-Ti rotor. The analysis of the gradients, collections of the fractions and quantification were performed as in the protocol for polysome profiling using crosslinking.
(DOC)
