# Supplementary figures and images for: The Deoxyhypusine Synthase Mutant dys1-1 Reveals the Association of eIF5A and Asc1 with Cell Wall Integrity
Source: PLoS One. 2013 Apr 1;8(4):e60140. doi: 10.1371/journal.pone.0060140 (PMC3613415; doi:10.1371/journal.pone.0060140)

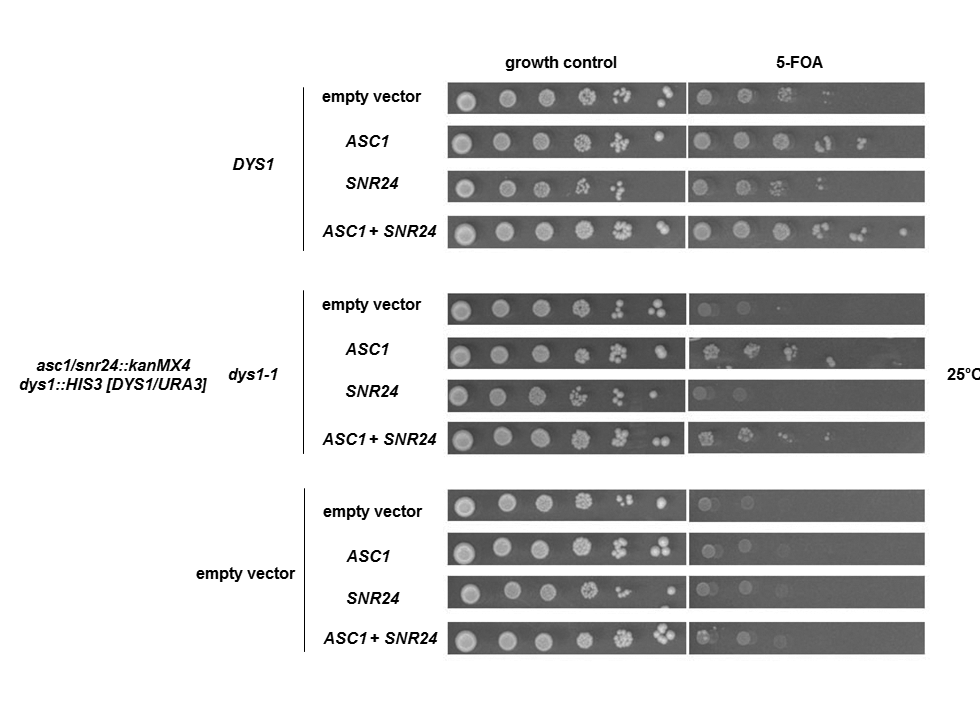

Supplement: Figure S1 — The dys1-1 mutant grows only in the presence of ASC1 . The indicated strains harboring DYS1, dys1-1 or empty vector were transformed with ASC1 (without SNR24), SNR24 alone, the entire ASC1 gene (ASC1+ SNR24) or the empty vector and plated onto medium not containing or containing 5-FOA and grown at 25°C for 3 days for plasmid shuffle. (TIF) [file pone.0060140.s001.tif]

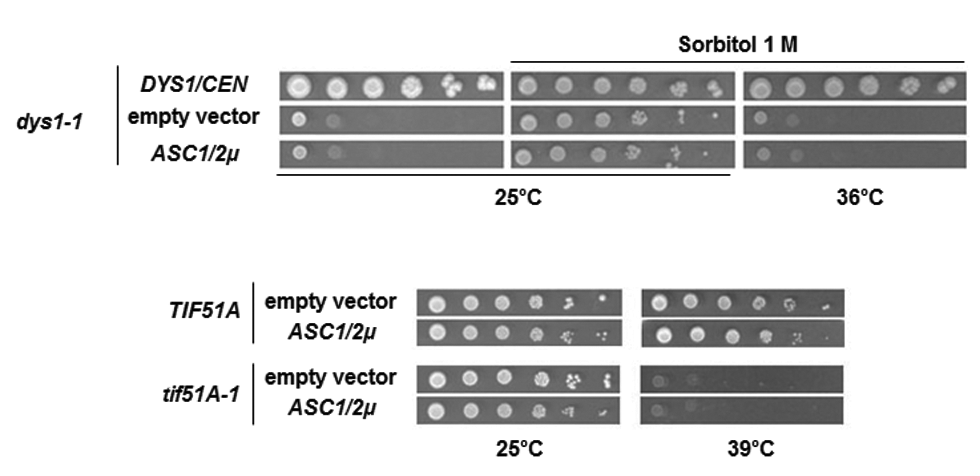

Supplement: Figure S2 — High-copy ASC1 did not suppress the dys1-1 and tif51A-1 growth defects. The dys1-1 and tif51A-1 mutants harboring ASC1 in high-copy plasmid (2μ) were grown at permissive and restrictive conditions for 3 days. (TIF) [file pone.0060140.s002.tif]

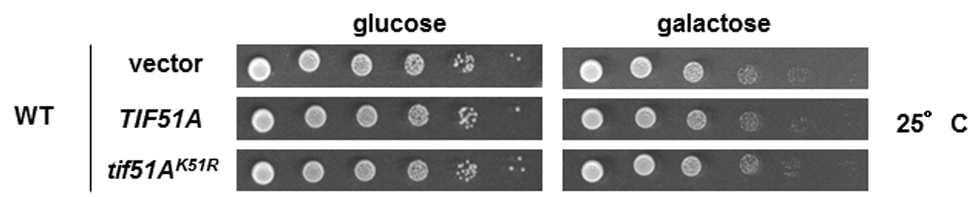

Supplement: Figure S3 — Overexpression of eIF5AK51R does not affect growth of wild type cells. Serial dilutions of wild type SVL272 transformed with vector pYES2, pSV975 (pYES2-TIF51A) and pSV976 (pYES2-tif51AK51R) were plated onto SC-ura supplemented with 2% glucose (growth control) or galactose (to induce heterologous eIF5A expression) and incubated at permissive temperature for 2 days. (TIF) [file pone.0060140.s003.tif]

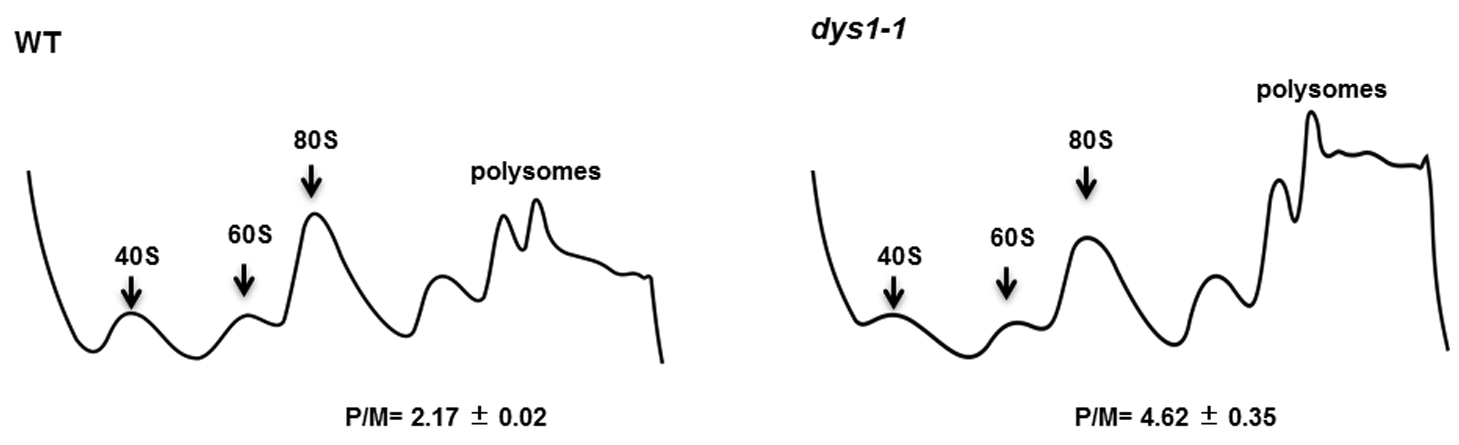

Supplement: Figure S4 — Polysome profiling of dys1-1 mutant after treatment with cycloheximide reveals the same defect found following formaldehyde crosslinking. Whole cell extracts (WCE) of the indicated strains, after treatment with cycloheximide, were fractionated through centrifugation in a sucrose density gradient. Optical scans (OD254nm) of the gradients are shown. The areas of the 80S and polysome peaks were compared to calculate the P/M ratio. (TIF) [file pone.0060140.s004.tif]
