# Supplementary material for: The Deoxyhypusine Synthase Mutant dys1-1 Reveals the Association of eIF5A and Asc1 with Cell Wall Integrity
Source: PLoS One. 2013 Apr 1;8(4):e60140. doi: 10.1371/journal.pone.0060140 (PMC3613415; doi:10.1371/journal.pone.0060140)
Supplement: Method S1 — Polysome profiling using cycloheximide. The cells from 200-mL cultures were grown to mid-log phase and treated with 10 µg/mL of cycloheximide for 5 min. The extracts were used for each sucrose gradient. Briefly, 20 A260nm units of cell lysates were layered onto 7–47% sucrose gradients containing 10 µg/mL of cycloheximide and centrifuged for 3 h at 39,000 rpm at 4°C in a Beckman SW41-Ti rotor. The analysis of the gradients, collections of the fractions and quantification were performed as in the protocol for polysome profiling using crosslinking. (DOC) [file pone.0060140.s005.doc]

**Supplementary Material and Methods and Figure legends**

**Polysome profiling using cycloheximide**

The cells from 200-mL cultures were grown to mid-log phase and treated with 10 μg/mL of cycloheximide for 5 min. The extracts were used for each sucrose gradient. Briefly, 20 A_260nm_ units of cell lysates were layered onto 7-47% sucrose gradients containing 10 μg/mL of cycloheximide and centrifuged for 3 h at 39,000 rpm at 4°C in a Beckman SW41-Ti rotor. The analysis of the gradients, collections of the fractions and quantification were preformed as in the protocol for polysome profiling using crosslinking.

**Supplementary Figure S1. The *dys1-1* mutant grows only in the presence of *ASC1*.** The indicated strains harboring *DYS1*, *dys1-1* or empty vector were transformed with *ASC1* (without *SNR24*), *SNR*24 alone, the entire *ASC1* gene (*ASC1* + *SNR24*) or the empty vector and plated onto medium not containing or containing 5-FOA and grown at 25°C for 3 days for plasmid shuffle.

**Supplementary Figure S2. High-copy *ASC1* did not suppress the *dys1-1* and *tif51A-1* growth defects.** The *dys1-1* and *tif51A-1* mutants harboring *ASC1* in high-copy plasmid (*2μ*) were grown at permissive and restrictive conditions for 3 days.

**Supplementary Figure S3.** **Overexpression of eIF5A^K51R^ does not affect growth of wild type cells.** Serial dilutions of wild type SVL272 transformed with vector pYES2, pSV975 (pYES2-*TIF51A*) and pSV976 (pYES2-*tif51A^K51R^*) were plated onto SC-ura supplemented with 2% glucose (growth control) or galactose (to induce heterologous eIF5A expression) and incubated at permissive temperature for 2 days.

**Supplementary Figure S4.** **Polysome profiling of *dys1-1* mutant after treatment with cycloheximide reveals the same defect found following formaldehyde crosslinking,.** Whole cell extracts (WCE) of the indicated strains, after treatment with cycloheximide, were fractionated through centrifugation in a sucrose density gradient. Optical scans (OD_254nm_) of the gradients are shown. The areas of the 80S and polysome peaks were compared to calculate the P/M ratio.
